# An investigation of the validity of the virtual spatial navigation assessment

**DOI:** 10.3389/fpsyg.2013.00852

**Published:** 2013-12-13

**Authors:** Matthew Ventura, Valerie Shute, Tim Wright, Weinan Zhao

**Affiliations:** ^1^College of Education, Florida State UniversityTallahassee, FL, USA; ^2^Department of Psychology, Florida State UniversityTallahassee, FL, USA

**Keywords:** environmental spatial ability, vista spatial ability, figural spatial ability

## Abstract

This correlational study investigated a new measure of environmental spatial ability (i.e., large scale spatial ability) called the virtual spatial navigation assessment (VSNA). In the VSNA, participants must find a set of gems in a virtual 3D environment using a first person avatar on a computer. The VSNA runs in a web browser and automatically collects the time taken to find each gem. The time taken to collect gems in the VSNA was significantly correlated to three other spatial ability measures, math standardized test scores, and choice to be in a STEM (science, technology, engineering, or math) career. These findings support the validity of the VSNA as a measure of environmental spatial ability. Finally, self-report video game experience was also significantly correlated to the VSNA suggesting that video game may improve environmental spatial ability. Recommendations are made for how the VSNA can be used to help guide individuals toward STEM career paths and identify weaknesses that might be addressed with large scale spatial navigation training.

## INTRODUCTION

Spatial ability has been shown to play a significant role in achievement in science, technology, engineering, and mathematics (STEM) disciplines. For instance, Wai et al. (2009) showed that spatial ability was a significant predictor of STEM degree attainment, even after controlling for mathematical and verbal skills. Thus a thorough understanding of spatial ability and how it can be improved should be considered paramount in understanding how to engage students in STEM related fields.

One way spatial ability can be improved is through playing action video games (e.g., Dorval and Pepin, 1986; Subrahmanyam and Greenfield, 1994; De Lisi and Wolford, 2002; Green and Bavelier, 2006; Feng et al., 2007; Spence et al., 2009; Uttal et al., 2012). For example, Feng et al. (2007) found that playing an action video game improved performance on a mental rotation task. After only 10 h of training with an action video game, subjects showed gains in spatial ability via mental rotation tasks, with females performing equal to males after training. Control subjects who played a non-action game showed no improvement. Recently, Uttal et al. (2012) conducted a meta-analysis of 206 studies investigating the effect of training on spatial ability. Of these 206 studies, 24 used video games to improve spatial ability. The effect size for video game training was 54 (SE = 0.12). Findings like these have been explained due to the visual-spatial requirements of 3D action games which may enhance spatial abilities (e.g., Feng et al., 2007). However, others have found a lack of transfer effects between action video game playing and basic cognitive functions and skills (e.g., Boot et al., 2008) and have raised questions regarding the methodology of studies that observe transfer (Boot et al., 2013; Kristjánsson, 2013).

Of particular importance in understanding the malleability of spatial ability is the distinction between figural, vista, and environmental related spatial abilities (Montello, 1993; Montello and Golledge, 1999). Figural spatial ability is small in scale relative to the body and external to the individual. It can be apprehended from a single viewpoint in both flat pictorial and 3D space (e.g., small, manipulatable objects). It is most commonly associated with tests such as mental rotation and paper folding tasks. Vista spatial ability is the ability to imagine oneself in different locations within a small space without locomotion. Vista spatial ability is useful when trying to image how the arrangement of objects will look from various perspectives (Hegarty and Waller, 2004). Environmental spatial ability is large in scale relative to the body and is useful in navigating around large spaces such as buildings, neighborhoods, and cities, and typically requires locomotion (see Montello, 1993; for a discussion of other scales of space). Environmental spatial ability may require a person to mentally construct a cognitive map, or internal representation of the environment (Montello and Golledge, 1999).

Specific processes in environmental spatial ability may result from the accumulation of three main types of knowledge of the environment: landmark, route, and configurational knowledge (Tolman, 1948; Siegel and White, 1975). First, landmark knowledge is acquired of perceptual objects through visual encoding. These perceived landmarks are then assimilated and are connected sequentially along a traversed path into route knowledge. Configurational knowledge is formed through the amassing of route knowledge, as a map-like representation of the environment is formed that allows for navigational inferences (Siegel and White, 1975). For example, new routes and distance and direction judgments can be formed as a result of a navigator’s configurational knowledge. The details of these environmental representations depend on a number of factors including the perception of environmental information, the speed in which the information is encoded, and how the information is maintained (Ittelson, 1973).

Existing measures of environmental spatial ability include map retracing, distance estimation, direction estimation (Hegarty et al., 2006), and self-report measures (Hegarty et al., 2002). Map retracing and distance and direction estimation (Hegarty et al., 2006) require a participant first to navigate through an environment (real world, 3D virtual environment, or first-person video). Afterwards, a person can be asked to (a) judge the distance among various features in the environment, (b) provide direction estimates among features in the environment, or (c) draw a 2D map of the environment. While these measures may seem distinct, factor analysis has revealed that these three measures were highly correlated and loaded on one factor, suggesting they measure a common ability (Hegarty et al., 2006). Additionally, factor analysis has shown measures based on virtual and video environments load on one factor (video factor) while measures based on real environments load on a second factor (real environment factor). The correlation between the two factors was high (*r* = 0.61) suggesting that the cognitive processes being used in virtual simulations are similar to the ones being used in real environments (Hegarty et al., 2006).

### THE PRESENT STUDY

Hegarty et al. (2006) proposed three main sources of variance in environmental spatial ability: (1) ability to encode spatial information from sensory experience, (2) ability to maintain a high quality internal representation of that information in memory, and (3) ability to perform spatial transformations in order to make inferences from this spatial information. In line with this theory, we developed the Virtual Spatial Navigation Assessment (VSNA). Advances in game development software now enable researchers with little programing experience to create virtual environments. These virtual environments are increasing being used to assess large scale spatial ability. For example, Herting and Nagel (2012) created a virtual water maze task to assess visuospatial memory. The VSNA requires a participant to explore a virtual 3D environment using a first person avatar on a computer. One significant difference between the VSNA and traditional measures of environmental spatial ability (e.g., map retracing) is that the VSNA collects data while a person is *in* the environment itself as opposed to collection of data *post hoc outside* of the environment. Measures (e.g., direction and distance estimation) based on one’s memory of an environment may be a source of construct irrelevant variance in the assessment of environmental spatial ability (Hegarty et al., 2006). Additionally, measurement outside of navigation in the environment requires individuals to make inferences that were not made within the environment (Montello et al., 2004). For example, the ability to point accurately to locations or estimate distances requires one to remember spatial configurations encoded in an environment. Assessing navigational performance in the task itself removes the additional burden of memory requirements that may contaminate the assessment of environmental spatial ability.

In each VSNA environment, a person must collect a set of brightly colored gems which are scattered throughout the environment. Participants need to complete the task twice for each environment. The first collection of gems is the training phase, which is intended to familiarize the person with the environment. The second collection of gems is the testing phase, which requires the person to obtain all the gems again as fast as possible. While no questions are explicitly asked about distance or direction between objects, the time to complete the VSNA require distance and direction estimation.

In this correlational study, we address two research questions centered around the validity of the VSNA as a measure of environmental spatial ability. The first question refers to how the VSNA relates to other measures of spatial ability. We compare the VSNA to a measure of figural spatial ability (mental rotation test, MRT; adapted from Vandenberg and Kuse, 1978), vista spatial ability (spatial orientation test, SOT; Hegarty and Waller, 2004) and a self-report measure of environmental spatial ability (SBSOD; Hegarty et al., 2002). In the MRT, participants view a 3D target figure and four test figures. The task is to determine which two test figures are correct rotations of the target figure as quickly and accurately as possible. The SOT requires the participant to make direction estimations from different perspectives relative to a 2D picture. For example, the person may be asked to give the direction of a car from the perspective as if the person is standing at a tree facing a traffic light. The degree to which a person can give the correct direction of objects from various perspectives is proposed to assess vista spatial ability. The SBSOD scale measures a person’s self-report belief about various navigation abilities in the real world (e.g., I don’t have a very good “mental map” of my environment, I enjoy reading maps). The SBSOD has been found to correlate (e.g., *r* = 0.44) with tests of spatial knowledge that involve orienting oneself within real-life environments (Hegarty et al., 2002). We also compare the VSNA to verbal and math scholastic aptitude test (SAT) scores since spatial ability has been shown to correlate to math achievement but not verbal achievement (e.g., Hegarty et al., 2006). Thus we expect to show divergent validity of the VSNA by showing it does not relate to verbal SAT scores.

Finally, regarding criterion related validity, we will investigate the relationship between the VSNA scores and choosing a STEM career path. Addressing this question expands on the work by Wai et al. (2009) showing that spatial ability predicts STEM degree attainment.

We make the following hypotheses regarding question one:

(1) *The VSNA should relate more to the SBSOD scale than the SOT and the MRT.* While the SOT and the MRT have been shown to relate to the SBSOD scale (e.g., Kozhevnikov and Hegarty, 2001), the VSNA should more accurately assess environmental spatial ability than the MRT or the SOT.

(2) *The VSNA will relate higher to math SAT scores than verbal SAT scores.* Spatial skills correlate to math achievement (e.g., Hegarty et al., 2006). Therefore the VSNA should relate more to math SAT scores than to verbal SAT scores.

(3) *The VSNA, SOT, and MRT will relate to STEM career path and achievement after controlling for gender, verbal and math ability (via SAT scores).* Spatial ability has been found to predict STEM degree attainment (Wai et al., 2009). Thus we expect to see a similar result for the VSNA as well as for the MRT and SOT.

The second question refers to the extent to which video game use influences environmental spatial ability (as measured by the VSNA). The question further addresses the malleability argument that video game use can impact spatial ability and specifically, environmental spatial ability. While this study focuses on correlational relationships, it is informative since it may show that even casual video game use can have a potential effect on environmental as well as vista and figural spatial ability.

Despite the large body of work investigating the role of video games on spatial ability, we are aware of only three studies that have specifically investigated the relationship between video game use and environmental spatial ability (Rehfeld et al., 2005; Schuster et al., 2008; Richardson et al., 2012). Schuster et al. (2008) found that video game experience correlated with college students’ ability to plan routes for unmanned vehicles in a 3D virtual simulation.

We make the following hypothesis regarding question two:

(4) *Video game use will relate to performance on all measures of spatial ability (figural, vista, environmental).* Higher video game use will be associated with in better spatial abilities compared to less video game use.

## MATERIALS AND METHODS

### PARTICIPANTS

323 undergraduate students (129 males, 194 females) enrolled in introductory psychology and education courses at a large southeastern state university volunteered to participate in the study for course credit.

### MEASURES

#### Virtual spatial navigation assessment

**FIGURE 1:**
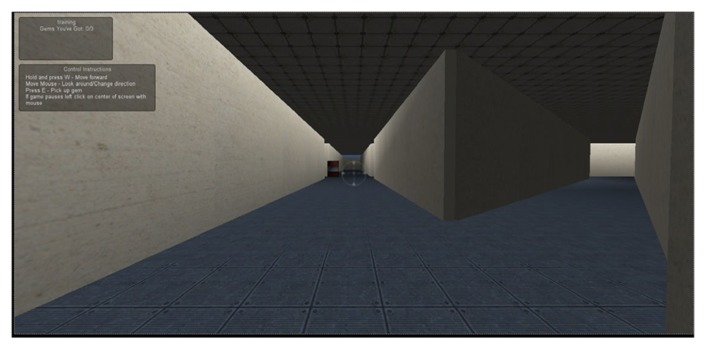
**Indoor environment in the VSNA**.

The VSNA was created in Unity, a free video game development software tool. In the VSNA, a participant explores a virtual 3D environment using a first person avatar on a computer. The avatar is controlled by a single key on the keyboard and the mouse. Pressing the key moves the avatar forward and the mouse controls the direction of the avatar. Participants are instructed that the goal is to collect three gems in an environment and return to the starting position. Participants first complete a short familiarization task that requires them to collect three gems in a small room. The VSNA consists of: (a) a small indoor environment consisting of halls (easy indoor), (b) a larger indoor environment (hard indoor), (c) a small outdoor environment (easy outdoor), and (d) a larger outdoor environment (hard outdoor). In each environment three gems are strategically located in the four environments so that an optimal path can be used to collect all the gems. In each environment the participant must collect the gems twice. The first collection is the training phase and the second collection is the testing phase. **Figure 1** displays a screenshot of the easy indoor environments.

The VSNA records the time taken to complete the training and testing phases per environment (i.e., time taken to collect the gems). The training phase is intended to measure one’s ability to *search* and *encode* information in the environment, while the testing phase is intended to measure one’s ability to *retrieve* and *apply* the encoded information. There is a 5 min time limit (per phase) in the easy indoor environment. The hard indoor and both outdoor environments each have a 10 min time limit per phase. If a person times out in a training phase, the participant skips the testing phase and goes to the next environment. The automated skip was done to keep the testing phase a recall task not a searching task. Lower score indicate higher environmental spatial ability. For ease of reading we reverse coded the VSNA scores so higher scores mean better performance.

#### The Santa Barbara sense of direction scale (Hegarty et al., 2002)

This test consists of 15 self-report items pertaining to environmental spatial ability (e.g., I am good at reading maps) that are rated on a five point likert scale. Higher score indicate higher environmental spatial ability.

#### Spatial orientation test (Hegarty and Waller, 2004)

This test consists of 12 questions requiring the participant to estimate locations of objects from different perspectives in one picture. In each item the participant is told to imagine looking at one object from a particular location in the picture and then point to a second location. An example item is as follows: *Imagine you are looking at the tree from the position of the cat, now point to the car*. The participant must draw the direction in a circle relative to an arrow in the circle that is always pointing to the 12 o’clock position. Each response is scored as a difference between the participant’s angle and the correct angle (ranges from 0° to 180°). Larger differences between a participant’s drawn angle and the correct angle indicate lower vista spatial ability. For ease of reading we reverse coded the SOT scores so higher difference scores mean greater vista spatial ability.

#### Mental rotation test (adapted from Vandenberg and Kuse, 1978)

In this test, participants view a three-dimensional target figure and four test figures. Their task is to determine which of the test figures are rotations of the target figure. The MRT has two correct answers for each of the 10 items. The total score is based on the total number of items where both correct objects are found. Higher score indicate higher figural spatial ability.

#### Video game use and VSNA-video game similarity

Participants answered one question about general video game use: How often do you play video games? 1 = not at all, 2 = about once a month, 3 = a few times a month, 4 = a few times a week, 5 = everyday, but for less than 1 h, 6 = every day for 1–3 h, 7 = every day for more than 3 h (Jackson et al., 2012). Additionally, participants were asked a question about the similarity between the VSNA and the video games they play: How similar was the navigation task to a video game you play (not at all, somewhat similar, similar, very similar, completely identical)?

### PROCEDURE

The study was conducted online in a web browser without supervision. Participants first reported their GPA, SAT scores, academic major, and completed the SBSOD scale. Then they completed the VSNA, the SOT, and the MRT. Finally, they completed some questions about video game use and usability of the VSNA. No tests were counterbalanced since we wanted to see how participants performed on the VSNA without the influence of fatigue from other spatial ability tests.

## RESULTS

**Table 1** displays the means and standard deviations of the SBSOD, times across the eight phases of the VSNA, the SOT, and MRT (listed in the order they were presented). Due to the difficult nature of the VSNA hard environments, and the study being unproctored, not all participants completed all tests. In some cases a participant timed-out of a training environment which results in the participant skipping its corresponding testing phase (as described in the VSNA measures section). In order to maximize power we still included participants in analysis who completed the training phase for each environment.

**Table 1:** Means and SDs of the SBSOD, VSNA phases, SOT, and MRT.

	N	**Mean**	**SD**
SBSOD	323	3.16	0.79
Easy indoor train^*^	323	132.69	55.59
Easy indoor test^*^	310	102.64	41.89
Hard indoor train^*^	322	206.64	115.66
Hard indoor test^*^	308	161.36	82.87
Easy outdoor train^*^	300	279.49	178.35
Easy outdoor test^*^	252	93.70	32.47
Hard outdoor train^*^	282	350.94	177.97
Hard outdoor test^*^	212	118.51	93.54
SOT	273	38.11	27.30
MRT	271	4.77	2.73

* Measured in seconds

Reliability was good for the SBSOD (α = 0.89), MRT (α = 0.76), and SOT (α = 0.87). Based on the high correlation between VSNA testing and training times (*r* = 0.61), we took the average score across training and testing. Easy and hard times were also highly related (*r* = 0.56) so we took the average score across easy and hard times to yield an indoor and outdoor VSNA score. While the correlation between indoor and outdoor environments was also high (*r* = 0.53), we report results for them separately since the sample size differs between the indoor and outdoor environments. Additionally, combining the time data across the indoor and outdoor environments could give an added advantage to participants who did not complete the outdoor task (i.e., give lower means to a person who did not complete the outdoor versus a person who did complete the outdoor).

We recoded students’ self-reported major into two categories: STEM related and non-STEM related. Examples of STEM related majors include: biology, engineering, computer science, and chemistry. Examples of non-STEM related majors include: English, education, business, communication, and history. Non-majors (*n* = 36) were excluded from the STEM major variable. **Table 2** displays the correlations between STEM career path (0 = non-STEM, 1 = STEM), gender (males = 0, females = 1), SAT math scores, MRT, SOT, and the indoor and outdoor VSNA scores (time data, where less time is better). GPA was omitted from **Table 2** since it did not relate to any spatial ability measures.

**Table 2:** Correlations (*r*) among gender, STEM major, SAT, spatial measures, and video game experience.

	Gender	STEM	SATm	SATv	SBSOD	MRT	SOT	Indoor	Outdoor
STEM	-0.12^*^
SATm	-0.17^**^	0.16^*^
SATv	0.05	0.10	0.62^**^
SBSOD	-0.33^**^	0.14^*^	0.17^**^	-0.01
MRT	-0.23^**^	0.10	0.24^**^	0.14	0.17^**^
SOT	-0.24^**^	0.08	0.24^**^	0.01	0.17^**^	0.45^**^
Indoor	-0.44^**^	0.22^**^	0.16^**^	0.02	0.37^**^	0.24^**^	0.18^**^
Outdoor	-0.37^**^	0.14^*^	0.15^*^	-0.04	0.19^**^	0.26^**^	0.18^**^	0.53^**^
VG use	-0.62^**^	0.03	0.19^**^	-0.00	0.18^**^	0.17^**^	0.29^**^	0.37^**^	0.33^**^

* p < 0.05; **p < 0.01; SATm, SAT math; VG use, video game experience

Regarding hypothesis one (i.e., VSNA should relate more to the SBSOD scale than the SOT and the MRT) both the indoor and outdoor VSNA scores significantly relate to the SBSOD, MRT, and the SOT. However, only the indoor VSNA scores appear to support hypothesis one: indoor VSNA scores are more highly correlated to the SBSOD (*r* = 0.37) relative to the MRT (*r* = 0.24) and the SOT (*r* = 0.18). The Steiger test (1980) was conducted to test if the VSNA indoor scores are significantly higher to the SBSOD versus the MRT and SOT (using a one-tailed test). The difference between the SBSOD (*r* = 0.37) and SOT (*r* = 0.18) is significant (*z* = 2.51, *p* < 0.05). The difference between SBSOD (*r* = 0.37) and MRT (*r* = 0.24) is significant (*z* = 1.74, *p* < 0.05). The VSNA indoor and outdoor times both account for unique variance in the SBSOD after controlling for MRT and SOT scores (*pr* = 0.36, *p* < 0.001; *pr* = 0.18, *p* < 0.001).

Regarding hypothesis two (i.e., VSNA will relate higher to math SAT scores than verbal SAT scores), all spatial ability measures related to SAT math scores. No spatial ability measures related to GPA or verbal SAT.

Regarding hypothesis three (i.e., VSNA, SOT, and MRT will relate to STEM major and achievement after controlling for gender, verbal and math ability), gender, SAT math scores, SBSOD, and both indoor and outdoor VSNA scores significantly relate to STEM majors. A hierarchical regression was run to predict STEM major. Predictors were entered in the following order: gender, math SAT scores, SBSOD scores, and finally VSNA indoor scores. Only VSNA indoor was a significant predictor of STEM interest after controlling for all other predictors (std β = 0.24, *p* < 0.01; *R*^2^ change = 0.04, *F*(1,219) = 10.13, *p* < 0.05). The same analysis was conducted entering VSNA outdoor scores last (gender, math SAT scores, SBSOD scores, VSNA outdoor scores) but the *R*^2^ change was not significant. GPA did not relate to any spatial ability measures for the STEM majors (*n* = 119).

Regarding hypothesis four (i.e., video game use will relate to performance on all measures of spatial ability), video game use significantly relates to the four spatial ability measures. However, after controlling for gender and video game similarity (for VSNA only), video game use only relates to SOT (*pr* = 0.21, *p* < 0.01) and VSNA indoor scores and (*pr* = 0.15, *p* < 0.05).

## DISCUSSION

Hypothesis one (i.e., VSNA should relate more to the SBSOD scale than the SOT and the MRT) was partially confirmed. We found that indoor VSNA scores had moderately highly correlations to SBSOD scores than to MRT and SOT scores. Both the VSNA indoor and outdoor scores accounted for unique variance in the SBSOD after controlling for MRT and SOT scores. These findings partially supports the construct validity of the VSNA as a measure of environmental spatial ability. We did not find evidence of construct validity for the outdoor VSNA scores (i.e., outdoor scores were more highly correlated to the MRT than to SBSOD). This finding may be due to the outdoor environments always being after the indoor environments which could cause fatigue effects on the outdoor environments. Finally, method effects (i.e., different task requirements) and well known psychometric issues related to self-report measures (e.g., social desirability) could be a reason why the correlation was not higher between the SBSOD and the VSNA.

Hypothesis two (i.e., VSNA will relate higher to math SAT scores than verbal SAT scores) was confirmed. We found that the VSNA, MRT, and SOT scores were all significantly related to math SAT scores and not verbal SAT scores. This is consistent with other research (e.g., Hegarty et al., 2006) that has shown a relation between mathematical and spatial abilities. This finding further supports the construct validity of the VSNA as a measure of spatial ability.

Hypothesis three (i.e., VSNA, SOT, and MRT will relate to STEM major and achievement after controlling for verbal and math ability) was partially supported. The VSNA indoor scores significantly correlated to being a STEM career path after controlling for gender, math SAT scores, and SBSOD scores (verbal SAT scores were not related to STEM career path). Thus we established criterion related validity of the VSNA. This finding extends the work by Wai et al. (2009) who showed that spatial ability was a significant predictor of STEM career path, even after controlling for math and verbal skills. However, we did not find the VSNA outdoor scores predicted STEM career path. This may be due to the lower number of participants who completed the outdoor VSNA. We also did not find that figural or vista spatial ability related to STEM career path. Thus environmental spatial ability may be a unique spatial ability separate from figural and vista ability that affects STEM career path. Additionally, no spatial ability measures related to GPA. Spatial ability may not give students an added academic advantage in STEM courses. However, the GPA variable was based on a variety of courses outside of STEM subjects. Future work research should investigate how environmental spatial ability relates to grades and performance in specific STEM courses.

Hypothesis four (i.e., video game use will relate to performance on all measures of spatial ability) was partially supported. Video game use was significantly correlated with the SOT and the indoor VSNA after controlling for gender. Importantly, the VSNA does not just assess one’s ability to play video games–video game use significantly relates to the VSNA after controlling for VSNA-video game similarity. The relation between video game use and the VSNA might be underestimated considering we only asked a broad question about video game use. Future work should consider using more detailed questions regarding video game use to further identify if specific video game use (e.g., 3D video games) relates more strongly to the VSNA.

A case can also be made that the VSNA-video game similarity question might not be sufficient to measure how similar the VSNA is to video games. For example, a particular video game player might see lots of differences between the VSNA and video games in general (e.g., lack of controls, gameplay options), while another video game player might see lots of similarities (e.g., first-person exploration in a 3D world). Future work should consider more detailed questions regarding the similarity between the VSNA and video games. However, the VSNA requires little motor control beyond skills learned by normal computer use (single button press with one hand and mouse control with the other hand). In this regard, the VSNA can be seen as a transfer task of environmental spatial ability independent from other video game play heuristics (e.g., effective use of controllers). These results are consistent with experimental evidence that video game use can improve spatial ability (e.g., Uttal et al., 2012). Thus exposure to video games may affect one’s ability to encode, store, retrieve, and apply environmental spatial information. Contrary to this theory, Richardson et al. (2012) found video game use was related to a pointing task after navigating through a virtual environment but not a pointing task after navigating through a real environment. However, Richardson et al. (2012) states the limitation of using pointing tasks to assess environmental spatial ability in that pointing tasks do not require actual navigation to targets. Thus it is possible that video game use does improve actual navigation performance *in* real environments (i.e., environmental spatial ability) but not to pointing performance *after* navigating through real environments.

Finally, the positive relation between video game use and environmental spatial ability also shows what lifestyle factors might indirectly affect interest in STEM (i.e., both the VSNA and SAT math scores relate to STEM major). While we did not find that video game use directly relates to STEM interest, video game use may indirectly affect interest in STEM by positively affecting environmental spatial ability and math ability.

Consistent with other work on spatial abilities (e.g., Spence et al., 2009), we found robust gender differences among the spatial ability measures. Females scored significantly worse on the SBSOD, SOT, MRT, and the VSNA (indoor and outdoor) compared to males. Follow up analysis revealed that after controlling for video game experience this gender effect was only eliminated for performance on the SOT. Future research should investigate how training can eliminate the gender gap in environmental spatial ability.

Looking forward, the VSNA could potentially be used for large scale assessment since it is scalable (i.e., run in a web browser) and quick to administer (as short as 10 min). This is important due to the growing number of studies suggesting the need to assess spatial ability in our education system (e.g., Wai et al., 2009; Uttal et al., 2012). There are many STEM related careers (e.g., engineering, medicine) and non-STEM careers (e.g., transportation, military, tourism) that require high levels of environmental spatial ability. These fields could benefit from having an assessment to be used for selection as well as intervention work. Additionally, the VSNA is a performance-based assessment not subject to social desirability effects. This gives it an advantage over traditional environmental spatial ability measures (e.g., SBSOD). Finally, assessment studies of environmental spatial ability using the VSNA can be covert since the gem finding activity does not explicitly cue participants about the purpose of the VSNA. This can be useful in situations where test anxiety could potentially affect the validity of the test.

This study cannot rule out the selection hypothesis that people with high environmental spatial ability may enjoy playing video games more than people with low environmental spatial ability. Future work should focus on experimental research investigating how using 3D simulations or video games can improve performance in the environmental spatial ability. Another limitation in this study was students completed all tests online without proctor supervisions. Results might have been more robust if participants were directed to stay on task throughout the session.

Another limitation of this study was we were not able to investigate the latent factorial structure of the VSNA. Given the limited time we had to run reach participant we could only investigate two levels of difficulty (easy vs. hard) in two distinct environments (indoor vs. outdoor). Future work should investigate creating “forms” of the VSNA that contain multiple isomorphic environments of similar difficulty (e.g., five outdoor nature environments). These forms can be compared to other forms that contain other features of the VSNA (e.g., five outdoor nature environments, five indoor environments, five outdoor urban environments). This design allows for confirmatory factor analysis and structural equation modeling. Additionally, counterbalancing and time spacing between forms should be implemented to control for any fatigue effects that may be occurring as a function of extended VSNA testing.

Finally, virtual environments do not provide any information to body-based senses (i.e., vestibular, proprioceptive) and thus may afford less detailed representations than real world environments (Waller et al., 2004; Richardson et al., 2012). However, Wan et al. (2009) provide evidence that participants still spatially update (e.g., remember locations of objects and landmarks) information in virtual environments much like in real environments. Future work should investigate how performance in the VSNA relates to real world navigation tasks.

## Conflict of Interest Statement

The authors declare that the research was conducted in the absence of any commercial or financial relationships that could be construed as a potential conflict of interest.
